# 抗肿瘤治疗的肺腺癌患者发生静脉血栓栓塞的情况及危险因素分析

**DOI:** 10.3779/j.issn.1009-3419.2023.102.22

**Published:** 2023-06-20

**Authors:** Xing CHEN, Weiping XIE, Chaoli YUE, Ting TANG, Yuchuyuan SUN, Kouying LIU

**Affiliations:** 210029 南京，南京医科大学第一附属医院呼吸与危重症医学科; Department of Respiratory Medicine, First Affiliated Hospital of Nanjing Medical University, Nanjing 210029, China

**Keywords:** 肺肿瘤, 静脉血栓栓塞, 抗肿瘤治疗, Lung neoplasms, Venous thromboembolism, Antitumor therapy

## Abstract

**背景与目的:**

静脉血栓栓塞（venous thromboembolism, VTE）是癌症患者最常见的并发症，也是癌症患者死亡的第二大原因。肺腺癌患者发生VTE的管理重点在于早期、及时发现危险因素。本研究旨在调查抗肿瘤治疗的肺腺癌患者VTE发生现状，探讨在抗肿瘤治疗过程中发生VTE的影响因素，为VTE的早期识别和筛查提供依据。

**方法:**

收集2019年12月-2021年5月于南京医科大学第一附属医院确诊为肺腺癌且行抗肿瘤治疗患者的临床资料，分别采用Cox单因素及多因素回归分析、Kaplan-Meier曲线和Log-rank检验比较独立危险因素影响VTE发生的差异。

**结果:**

Cox回归单因素及多因素分析显示，VTE病史、靶向药物治疗、放疗是抗肿瘤治疗肺腺癌患者VTE发生的危险因素（P<0.05）。Kaplan-Meier曲线和Log-rank检验比较独立危险因素发现VTE病史、靶向治疗、放疗患者的VTE发生率更高（P<0.05）。

**结论:**

有VTE病史、放疗、靶向治疗是VTE发生的高危因素，对于这类影响因素的早期识别和筛查有助于今后临床实践中VTE的防控和管理。

## Abstract (en)

**Background and objective:**

Venous thromboembolism (VTE) as the most common cancer-associated complication has become the second death-causing reason among cancer patients. The management of VTE in patients with lung adenocarcinoma should focus on early and timely detection of risk factors. The aim of the study is to investigate the current situation of VTE in patients with lung adenocarcinoma treated with anti-tumor therapy and then explore the risk factors associated with the occurrence of VTE during anti-tumor therapy for early detection and screening of VTE.

**Methods:**

The present study included patients diagnosed as lung adenocarcinoma undergoing anti-tumor therapy in First Affiliated Hospital of Nanjing Medical University between December 2019 and May 2021. The risk factors were identified via univariate and multivariate Cox analysis. The incidence of independent risk factors were investigated through Kaplan-Meier curves combined with Log-rank test.

**Results:**

The results of univariate and multivariate Cox regression showed that history of VTE, targeted therapy and radiotherapy were risk factors for VTE in patients with lung adenocarcinoma treated with anti-tumor therapy (P<0.05). Furthermore, the results of Kaplan-Meier curves and Log-rank tests indicated the incidences of VTE in patients with history of VTE, targeted therapy and radiotherapy were higher (P<0.05).

**Conclusion:**

History of VTE, radiotherapy and targeted therapy are found as independent risk factors for the occurrence of VTE, which should be identified and monitored for reduction of VTE incidence.

静脉血栓栓塞（venous thromboembolism, VTE）是指由于血液异常凝结、堵塞血管所引起的静脉回流受阻性疾病，包括深静脉血栓形成（deep venous thromboembolism, DVT）和肺血栓栓塞症（pulmonary thromboembolism, PE）两种类型^[1]^。VTE是癌症患者最常见的并发症，也是癌症患者死亡的第二大原因，其发病率为15%，死亡率为12.7%^[2,3]^。VTE会导致一系列的不良临床结局，如加速病情恶化；延长住院时间、增加住院费用；缩短预期生存时间，增加痛苦心理；增加出血风险等^[2,4]^。肺腺癌是非小细胞肺癌（non-small cell lung cancer, NSCLC）中VTE发生率最高的病理分型^[5]^。由于较多肺腺癌患者就诊时已属晚期，手术机会小，因此肺腺癌的主要抗肿瘤治疗方案包括放疗、化疗、靶向治疗、免疫治疗^[6]^。研究^[7⇓-9]^发现，抗肿瘤治疗会导致VTE的发生率增加。早期、及时识别并控制VTE的危险因素有助于临床实践中预防肺腺癌患者发生VTE或者改善肺腺癌合并VTE患者的生存结局。

目前，大多数肺腺癌合并VTE的相关研究尚未考虑抗肿瘤治疗对VTE发生的影响^[10]^。本研究旨在探索肺腺癌患者在抗肿瘤治疗过程中VTE的发生率及影响因素，有助于临床医疗及护理人员在临床实践中预防及管理VTE。现报道如下。

## 1 资料与方法

### 1.1 研究对象

本研究以2019年12月-2021年5月于南京医科大学第一附属医院确诊为肺腺癌并行抗肿瘤治疗的患者为研究对象。纳入标准：（1）经组织病理学或细胞学确诊为肺腺癌；（2）接受过至少1次多普勒血管超声检查或肺动脉计算机断层扫描（computed tomography, CT）血管成像；（3）肝肾功能及骨髓造血功能未发生异常；（4）年龄<85岁。排除标准：（1）原发于其他部位的腺癌转移至肺；（2）血液系统异常；（3）激素或免疫检查点抑制剂治疗史；（4）伴有严重感染、心功能异常等；（5）自身免疫性疾病；（6）合并其他类型肿瘤；（7）临床资料缺失者。本研究经医院伦理委员会审核并批准通过（No.2020583），患者及家属均知情同意并自愿参加。随访截止时间为2023年2月。

### 1.2 研究方法

#### 1.2.1 研究工具

##### 1.2.1.1 临床资料收集

由研究者通过文献回顾自行设计问卷调查表，内容条目涵盖：（1）人口学基线资料：性别、年龄、身体质量指数（body mass index, BMI）、吸烟史、饮酒史；（2）临床基本相关资料：肺腺癌原发部位，肺癌根治术，肿瘤分期，转移部位，合并疾病（高血压、糖尿病、心脑血管疾病、肺部疾病、肝脏疾病、肾脏疾病、甲状腺疾病），治疗方案（化疗、放疗、靶向治疗、免疫治疗），治疗周期，VTE病史，血栓弹力图，凝血指标等。

##### 1.2.1.2 年龄校正Charlson合并症指数（age-adjusted Charlson comorbidity index, aCCI）

aCCI由Charlson等^[11]^于1994年制定，其可量化患者所患疾病数目及严重程度并进行评分；目前作为最为常见的合并症评价体系之一，aCCI被广泛运用于疾病（包括癌症）的预后预测^[12]^。aCCI由16种常见的共患疾病加上对应年龄分组评分得出最终得分。得分越高，预后越差，生存率越低。

##### 1.2.1.3 血栓的确诊方法

符合中华医学会外科学分会血管外科学组颁布的《深静脉血栓形成的诊断和治疗指南》和/或中华医学会呼吸病学分会肺栓塞与肺血管病学组颁布的《肺血栓栓塞症诊治与预防指南》，经双下肢静脉彩色多普勒超声检查或肺动脉造影检查等确诊为VTE。

#### 1.2.2 资料收集方法

由2名呼吸科工作时间超过1年的护士通过IH系统搜索出院诊断关键词“肺腺癌”，根据肺癌诊断后的3个月-6个月VTE的发病风险较高的临床特点^[13]^，选择其中行抗肿瘤治疗以及随诊时间≥3个月的患者，并从中剔除第一次行抗肿瘤治疗时入院或出院诊断包含“DVT/肺栓塞/PE/静脉血栓栓塞症”的患者。记录患者的相关资料。随访期间以患者结束抗肿瘤治疗、并发VTE、死亡作为终点事件。收集完成后，由1名呼吸科工作时间超过3年的护士进行资料整理，确保资料的真实性和可靠性。最终将发生VTE的32例肺腺癌患者纳入VTE组，未发生VTE的193例纳肺腺癌患者纳入非VTE组。

### 1.3 统计学分析

采用SPSS 20.0进行分析。计数资料采用例数及对应百分比进行描述，组间比较采用χ^2^检验或Fisher确切概念法分析；计量资料应用均数±标准差（Mean±SD）或中位数（Median）进行描述，组间比较采用t检验或Mann-Whitney U检验分析。采用Cox比例风险模型进行单因素回归分析，对差异具有统计学意义的因素进一步行多因素分析，筛选出肺腺癌患者抗肿瘤治疗后发生VTE的独立危险因素。同时，采用Kaplan-Meier曲线和Log-rank检验比较独立危险因素导致VTE发生率的差异。P<0.05代表差异有统计学意义。

## 2 结果

### 2.1 VTE发生率

本研究共纳入肺腺癌患者225例，其中非VTE组193例，VTE组32例。抗肿瘤治疗的肺腺癌患者的VTE发生率为14.22%。

### 2.2 一般资料

非VTE组女性84例，男性109例，平均年龄（59.51±9.16）岁。VTE组女性13例，男性19例，平均年龄（60.47±8.59）岁。非VTE组BMI为（23.81±3.19）kg/m^2^，而VTE组为（24.01±3.58）kg/m^2^。非VTE组具有吸烟史患者56例，饮酒史患者22例，VTE组具有吸烟史患者11例，饮酒史患者7例。纳入研究的合并疾病及基于aCCI的量化分析在VTE组和非VTE组的组间比较结果无明显差异（P>0.05）。其中非VTE组无VTE病史者，而VTE组中伴有VTE病史者3例，差异有统计学意义（P<0.05）。其中非VTE组中位随访时间为126 d，VTE组中位随访时间为123 d，差异无统计学意义（P>0.05）（表1）。

**表1:** 225例肺腺癌患者临床特征的单因素分析

Variables	Total (n=225)	VTE		P
		Negative (n=193)	Positive (n=32)	
Gender				0.76
Female	97 (43.11%)	84 (43.52%)	13 (40.62%)	
Male	128 (56.89%)	109 (56.48%)	19 (59.38%)	
Age (yr)				0.58
Mean±SD	59.65±9.07	59.51±9.16	60.47±8.59	
BMI (kg/m^2^)				0.75
Mean±SD	23.84±3.24	23.81±3.19	24.01±3.58	
Smoking history				0.54
Without	158 (70.22%)	137 (70.98%)	21 (65.63%)	
With	67 (29.78%)	56 (29.02%)	11 (34.37%)	
Alcohol abuse				0.10
Without	196 (87.11%)	171 (88.60%)	25 (78.13%)	
With	29 (12.89%)	22 (11.40%)	7 (21.87%)	
Hypertension				0.09
Without	162 (72.00%)	143 (74.09%)	19 (59.38%)	
With	63 (28.00%)	50 (25.91%)	13 (40.62%)	
Diabetes				0.35
Without	200 (88.89%)	170 (88.08%)	30 (93.75%)	
With	25 (11.11%)	23 (11.92%)	2 (6.25%)	
Cardiovascular diseases				0.62
Without	214 (95.11%)	183 (94.82%)	31 (96.88%)	
With	11 (4.89%)	10 (5.18%)	1 (3.12%)	
Cerebrovascular diseases				0.34
Without	209 (92.89%)	178 (92.23%)	31 (96.88%)	
With	16 (7.11%)	15 (7.77%)	1 (3.12%)	
Pulmonary diseases				0.20
Without	214 (95.11%)	185 (95.85%)	29 (90.62%)	
With	11 (4.89%)	8 (4.15%)	3 (9.38%)	
Hepatic disease				0.37
Without	217 (96.44%)	187 (96.89%)	30 (93.75%)	
With	8 (3.56%)	6 (3.11%)	2 (6.25%)	
Kidney disease				0.68
Without	224 (99.56%)	192 (99.48%)	32 (100.00%)	
With	1 (0.44%)	1 (0.52%)	0 (0.00%)	
Thyroid diseases				0.56
Without	223 (99.11%)	191 (98.96%)	32 (100.00%)	
With	2 (0.89%)	2 (1.04%)	0 (0.00%)	
aCCI (points)				0.89
Median (Q1, Q3)	2 (1, 3)	2 (1, 3)	2 (1, 2)	
VTE history				<0.05
Without	222 (98.67%)	193 (100.00%)	29 (90.63%)	
With	3 (1.33%)	0 (0.00%)	3 (9.37%)	
Follow-up period (d)				0.49
Median (Q1, Q3)	125.3 (84, 189)	126 (105, 210)	123 (42, 194)	

VTE: venous thromboembolism; BMI: body mass index; aCCI: age-adjusted Charlson comorbidity index.

### 2.3 实验室检查

收集两组患者的血小板、凝血指标[活化部分凝血活酶时间（activated partial thromboplastin time, APTT）、血浆凝血酶原时间（prothrombin time, PT）、血浆纤维蛋白原（fibrinogen, FIB）、国际标准化比值（international normalized ratio, INR）、D-二聚体]及血栓弹力图，结果显示两组间差异无统计学意义（P>0.05）（表2）。

**表2:** 225例肺腺癌患者实验室检查的单因素分析

Variables	Total (n=225)	VTE		P
		Negative (n=193)	Positive (n=32)	
Thrombelastogram				0.13
Normal	212 (94.22%)	180 (93.26%)	32 (100.00%)	
Abnormal	13 (5.78%)	13 (6.74%)	0 (0.00%)	
Platelet (×10^9^/L)				0.74
Normal	207 (92.00%)	177 (91.71%)	30 (93.76%)	
Higher than normal	13 (5.78%)	12 (6.22%)	1 (3.12%)	
Lower than normal	5 (2.22%)	4 (2.07%)	1 (3.12%)	
APTT (s)				0.77
Normal	195 (86.67%)	166 (86.01%)	29 (90.63%)	
Higher than normal	20 (8.89%)	18 (9.33%)	2 (6.25%)	
Lower than normal	10 (4.44%)	9 (4.66%)	1 (3.12%)	
PT (s)				0.41
Normal	221 (98.22%)	189 (97.93%)	32 (100.00%)	
Abnormal	4 (1.78%)	4 (2.07%)	0 (0.00%)	
FIB (g/L)				0.61
Normal	169 (75.11%)	147 (76.17%)	22 (68.75%)	
Higher than normal	47 (20.89%)	39 (20.21%)	8 (25.00%)	
Lower than normal	9 (4.00%)	7 (3.62%)	2 (6.25%)	
PT-INR				0.68
Normal	224 (99.56%)	192 (99.48%)	32 (100.00%)	
Abnormal	1 (0.44%)	1 (0.52%)	0 (0.00%)	
D-D (mg/L)				0.44
Normal	127 (56.44%)	112 (58.03%)	15 (46.88%)	
Abnormal	98 (43.56%)	81 (41.97%)	17 (53.12%)	

APTT: activated partial thromboplastin time; PT: prothrombin time; FIB: fibrinogen; PT-INR: PT-international normalized ratio; D-D: D-Dimer.

### 2.4 肿瘤相关观察指标

两组肺腺癌患者的肿瘤原发部位、肿瘤分期、肺内转移、淋巴结转移和远处转移情况无显著差异（P>0.05）。两组患者行肺癌根治术者，分别为非VTE组75例、VTE组8例，差异无统计学意义（P>0.05）。将接受外科治疗患者和未接受外科治疗的患者进行组间比较，VTE发生率差异无统计学意义（P>0.05）。

患者接受不同方案及周期的化疗，非VTE组及VTE组中位化疗周期均为126 d（P>0.05）。非VTE组接受放疗患者13例（6.74%），而VTE组共6例（18.75%），组间比较差异明显（P<0.05）。

非VTE组口服靶向药患者107例（55.44%），VTE组24例（75.00%），差异具有统计学意义（P<0.05）。此外，非VTE组接受免疫治疗共41例（21.24%），VTE组接受免疫治疗共6例（18.75%），中位免疫治疗周期分别为126 d及136.5 d，差异无统计学意义（P>0.05）（表3）。

**表3:** 225例肺腺癌患者肿瘤相关观察指标的单因素分析

Variables	Total (n=225)	VTE		P
		Negative (n=193)	Positive (n=32)	
Site				0.32
Left	89 (39.56%)	73 (37.82%)	16 (50.00%)	
Right	132 (58.67%)	117 (60.62%)	15 (46.88%)	
Both	4 (1.77%)	3 (1.56%)	1 (3.12%)	
Clinical stage				0.49
I-II	53 (23.56%)	47 (24.35%)	6 (18.75%)	
III-IV	172 (76.44%)	146 (75.65%)	26 (81.25%)	
Pulmonary metastasis				0.90
Without	181 (80.44%)	155 (80.31%)	26 (81.25%)	
With	44 (19.56%)	38 (19.69%)	6 (18.75%)	
Lymph node metastasis				0.36
Without	72 (32.00%)	64 (33.16%)	8 (25.00%)	
With	153 (68.00%)	129 (66.84%)	24 (75.00%)	
Distant metastasis				0.24
Without	99 (44.00%)	88 (45.60%)	11 (34.38%)	
With	126 (56.00%)	105 (54.40%)	21 (65.62%)	
Surgery				0.13
Without	142 (63.11%)	118 (61.14%)	24 (75.00%)	
With	83 (36.89%)	75 (38.86%)	8 (25.00%)	
Pulmonary infection				0.70
Without	214 (95.11%)	184 (95.34%)	30 (93.75%)	
With	11 (4.89%)	9 (4.66%)	2 (6.25%)	
Radiotherapy				<0.05
Without	206 (91.56%)	180 (93.26%)	26 (81.25%)	
With	19 (8.44%)	13 (6.74%)	6 (18.75%)	
Targeted therapy				<0.05
Without	94 (41.78%)	86 (44.56%)	8 (25.00%)	
With	131 (58.22%)	107 (55.44%)	24 (75.00%)	
Immunotherapy				0.75
Without	178 (79.11%)	152 (78.76%)	26 (81.25%)	
With	47 (20.89%)	41 (21.24%)	6 (18.75%)	
Pemetrexed				0.23
Without	22 (9.78%)	17 (8.81%)	5 (15.63%)	
With	203 (90.22%)	176 (91.19%)	27 (84.37%)	
Paclitaxel				0.79
Without	166 (73.78%)	143 (74.09%)	23 (71.88%)	
With	59 (26.22%)	50 (25.91%)	9 (28.12%)	
Platinum				0.86
Without	23 (10.22%)	20 (10.36%)	3 (9.38%)	
With	202 (89.78%)	173 (89.64%)	29 (90.62%)	
Period of chemotherapy (d)				0.37
Median (Q1, Q3)	126 (84, 189)	126 (84, 189)	126 (84, 168)	
Period of immunotherapy (d)				0.50
Median (Q1, Q3)	126 (63, 237)	126 (63, 231)	136.5 (84, 283.5)	

### 2.5 VTE发生危险因素的终点事件率比较

采用Kaplan-Meier曲线和Log-rank检验分别比较危险因素（VTE病史、靶向治疗、放疗）导致VTE发生率的差异。结果显示既往发生过VTE的患者再次发生VTE的风险高于无VTE病史的患者（HR=5.08, 95%CI: 1.55-16.69, P<0.05），行放疗的患者发生VTE的风险高于未进行放疗的患者（HR=1.41, 95%CI: 1.08-1.93, P<0.05），行靶向治疗的患者发生VTE的风险高于未进行靶向治疗的患者（HR=2.20, 95%CI: 1.05-4.63, P<0.05）（图1-图3）。

**图1:**
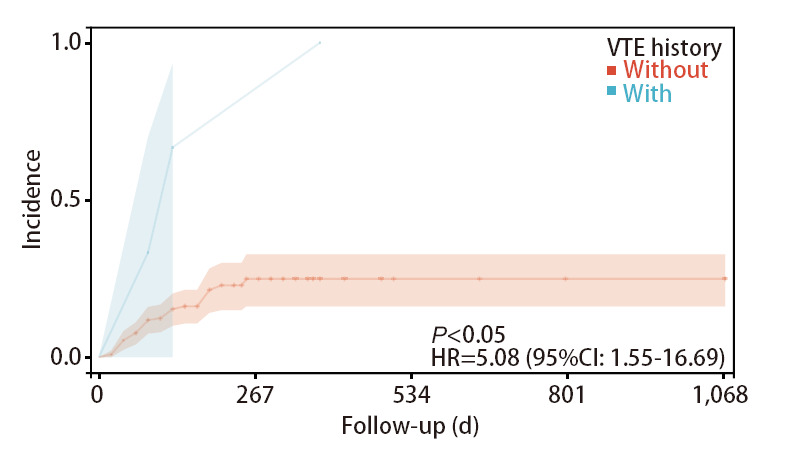
VTE病史患者与非VTE病史患者发生VTE的发生率曲线

**图2:**
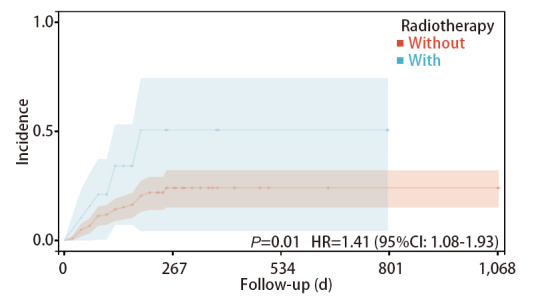
进行放疗的患者与未进行放疗的患者发生VTE的发生率曲线

**图3:**
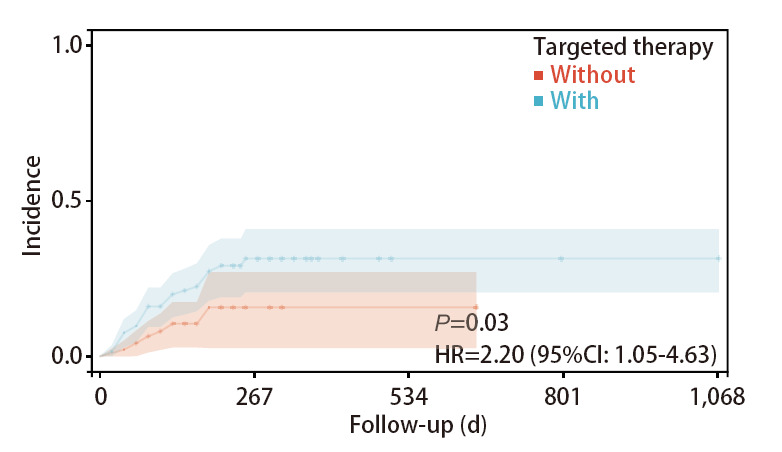
使用靶向药物治疗的患者与未使用靶向药物治疗的患者发生VTE的发生率曲线

### 2.6 VTE发生危险因素的Cox回归分析

首先，以患者在随访期间发生VTE作为结局指标，所有可能发生VTE的危险因素进行单因素Cox回归分析，结果显示，VTE病史、靶向治疗、放疗的差异有统计学意义（P<0.05）。随后，通过多因素Cox回归分析进一步显示，既往发生过VTE的患者再次发生VTE的风险高于无VTE病史的患者（HR=302.18, 95%CI: 21.65-495.53, P<0.05）。行放疗的患者发生VTE的风险高于未进行放疗的患者（HR=2.69, 95%CI: 1.10-6.55, P<0.05）。行靶向药物治疗的患者发生VTE的风险高于未进行靶向药物治疗的患者（HR=1.86, 95%CI: 1.50-2.73, P<0.05）（表4）。

**表4:** 腺癌抗肿瘤治疗患者发生静脉血栓栓塞的Cox回归分析

Characteristics	Univariate Cox regression analysis				Multivariate Cox regression analysis		
	HR	P	95%CI		HR	P	95%CI
Gender	1.02	0.95	0.50-2.07				
Age (yr)	1.01	0.74	0.97-1.05				
BMI (kg/m^2^)	1.02	0.69	0.92-1.14				
Smoking history	1.23	0.58	0.59-2.55				
Alcohol abuse	1.77	0.18	0.76-4.08				
Clinical stage	1.20	0.68	0.49-2.94				
Hypertension	1.75	0.12	0.87-3.55				
Diabetes	0.52	0.37	0.12-2.18				
Cardiovascular diseases	0.64	0.66	0.09-4.65				
Cerebrovascular diseases	0.37	0.32	0.05-2.69				
Pulmonary diseases	2.08	0.23	0.63-6.83				
Hepatic disease	2.18	0.29	0.52-9.16				
Kidney disease	0.00	1.00	0.00-Inf				
Thyroid diseases	0.00	1.00	0.00-Inf				
aCCI	0.95	0.73	0.71-1.27				
VTE history	298.67	0.00	15.65-495.53		302.18	<0.05	21.65-495.53
Site	0.63	0.20	0.31-1.28				
Pulmonary metastasis	0.89	0.79	0.36-2.15				
Lymph node metastasis	1.36	0.45	0.61-3.04				
Distant metastasis	1.39	0.38	0.67-2.89				
Surgery	0.60	0.22	0.27-1.34				
Pulmonary infection	1.17	0.83	0.28-4.91				
Radiotherapy	2.58	0.04	1.06-6.27		2.69	<0.05	1.10-6.55
Targeted therapy	1.79	0.03	1.38-2.62		1.86	<0.05	1.50-2.73
Immune therapy	1.74	0.16	0.80-3.77				
Pemetrexed	0.60	0.29	0.23-1.55				
Paclitaxel	1.01	0.98	0.47-2.18				
Platinum	1.19	0.78	0.36-3.9				
Thrombelastogram	0.00	1.00	0.00-Inf				
Platelet (×10^9^/L)	0.49	0.49	0.07-3.61				
APTT (s)	0.65	0.56	0.16-2.74				
PT (s)	0.00	1.00	0.00-Inf				
FIB (g/L)	2.02	0.34	0.47-8.61				
PT-INR	0.00	1.00	0.00-Inf				
D-D (mg/L)	1.39	0.35	0.69-2.79				

Inf: infimum.

## 3 讨论

近年来除了放疗、化疗等基础治疗外，靶向治疗和免疫治疗也成为了肺腺癌患者的主要治疗方式。在癌症治疗取得重大进展的同时，VTE的发生风险也有所增加。而我国暂未有关于肺腺癌抗肿瘤治疗患者VTE发生情况的相关报道。本研究结果显示，肺腺癌患者的VTE发生率为14.22%，高于既往相关研究^[13,14]^结果，可能与研究对象性质不同有关。既往研究选择未进行过抗肿瘤治疗的患者队列^[14]^，而本研究中纳入的是行抗肿瘤治疗的患者，考虑到化疗是引发VTE的危险因素，这可能是本研究中VTE发生率较高的原因之一。此外，既往研究^[13]^中纳入多种类型的肺恶性肿瘤，而本研究中仅纳入肺腺癌患者，恶性程度较其他类型肿瘤高，作为VTE的危险因素之一，可能导致本研究VTE发生率升高。肺腺癌患者肿瘤细胞可以释放出一些促凝物质，如凝血酶原激活物、组织因子等，这些物质会刺激血液凝结过程，从而导致VTE形成^[3]^。VTE的出现对患者的预后带来诸多不利。有研究^[15]^发现，约有20%的血栓患者因PE而死亡。因此识别抗肿瘤治疗的肺腺癌患者发生VTE的危险因素显得尤为重要。

本研究中，经过回归分析以及生存分析发现VTE病史与VTE发生有显著的相关性。且与非VTE组相比，存在VTE病史的患者，其VTE发生率远高于非VTE组。恶性肿瘤本身就可通过破坏机体正常的凝血-纤溶反应，使患者处于高凝状态；同时合并VTE病史的患者可能存在遗传性凝血功能缺陷或者获得性血栓形成相关高危因素，更易于VTE形成^[16]^。Chee等^[17]^研究认为，活动性癌症是VTE复发的主要预测因子，且脑癌、肺癌、其他IV期癌症与复发风险增加相关，其中10年累积VTE复发率为28.6%。既往VTE病史是VTE再发的独立危险因素，具有一定的临床意义。但既往多数文献的研究对象为发生VTE的患者，活动性肿瘤作为调查内容进行分析。而本研究以抗肿瘤治疗的肺腺癌患者为研究对象，并首次发现VTE病史是抗肿瘤治疗的肺腺癌患者发生VTE的独立危险因素。因此，对于有VTE病史的肺腺癌患者，临床医疗及护理人员需要进行VTE的早期预防和管理，如评估VTE的早期症状、关注实验室检查等，必要时指导患者使用抗凝药物治疗。

在本研究中，放疗是抗肿瘤治疗过程中的肺腺癌患者发生VTE的危险因素，发生率为18.75%。指南建议中晚期肺腺癌患者接受放化疗联合的标准治疗方案^[18]^。放疗通过电离辐射抑制肿瘤生长，可进一步显著延长晚期肺腺癌患者的生存，甚至部分晚期患者可获得治愈。但局部放疗也可能会造成包括疲劳、骨髓抑制在内的多种不良反应^[19,20]^。谢瑞杰等^[21]^基于213例恶性肿瘤患者的研究，使用多因素回归分析发现，放疗是肿瘤患者发生VTE的独立危险因素。Temraz等^[8]^纳入2013年-2015年1,355例接受放疗的肿瘤患者，其中9.1%的放疗患者发生VTE，经过竞争因素模型分析后发现放疗是VTE风险增加的危险因素。此外，Daguenet等^[22]^对401例放疗患者的分析发现，约2%的患者在治疗后6个月左右发生VTE。目前已有较多学者对放疗导致VTE发生的机制进行了探索，但其具体机制尚不明确。放疗产生的电离辐射，可能会导致血管内皮细胞释放促凝剂，并通过影响蛋白C的激活及其与血栓调节蛋白的相互作用，进而使抗凝系统异常，导致血栓形成^[23]^。因此，对于定期行放疗的肺腺癌患者，临床医疗及护理人员不仅要观察其是否出现疲乏、食欲减退、骨髓抑制、皮肤口腔黏膜破损等常见的放疗副反应，也应当观察患者是否出现下肢肿胀、疼痛等VTE相关症状。指导患者在病情允许下多饮水，适当运动，以预防VTE的发生。

本研究中VTE组使用靶向药物治疗比例为75.00%，非VTE组为55.44%，显示靶向治疗是肺腺癌患者VTE发生的独立危险因素。靶向治疗是近年来新兴的一种肿瘤治疗方式，其通过干扰肿瘤细胞增生所需的特定分子，达到抗癌的目的。近年来表皮生长因子受体酪氨酸激酶抑制剂（epidermal growth factor receptor-tyrosine kinase inhibitors, EGFR-TKIs）（如吉非替尼、厄洛替尼等）以及血管内皮生长因子（vascular endothelial growth factor, VEGF）-TKIs（贝伐珠单抗）、重组人血管内皮抑制素已成为晚期NSCLC不可或缺的治疗手段。既往研究^[24]^显示，相较于传统放化疗，靶向治疗在提高恶性肿瘤患者生存结局的同时，可降低不良反应的发生率，提高患者生活质量。其中，EGFR-TKIs常见的不良反应包括乏力、腹泻、皮疹、黏膜炎、甲沟炎等，罕见不良反应包括肝功能损害和间质性肺疾病^[25]^。而VEGF-TKIs的常见不良反应包括高血压、间质性肺疾病和胃肠道出血等^[26]^。然而，其中对于靶向药物是否造成血管不良反应的研究相对较少。2022年，Moik等^[27]^报道，VEGF-TKIs、EGFR-TKIs、CDK4/6抑制剂和第二代BCR-ABL抑制剂均具有促血栓形成的作用。Lorenzi等^[28]^对126例服用第三代EGFR-TKIs（奥希替尼）的NSCLC患者进行了随访研究，发现VTE发生率为7.9%，显著高于既往报道。多数服用靶向药物的NSCLC患者会选择在门诊进行随访，这局限了临床医疗及护理人员对患者的宣教和病情观察，往往患者就诊时已出现靶向治疗的不良反应。因此，临床医疗及护理人员不仅应对住院患者做好治疗及监测工作，也应制定针对门诊患者的疾病、药物的护理计划。

综上，本研究通过分析发现VTE病史、放疗、靶向治疗是肺腺癌患者发生VTE的独立危险因素。因此临床医疗及护理人员可针对肺腺癌在抗肿瘤治疗过程中发生VTE的影响因素进行早期评估及筛查，并及时采取干预措施。本研究的纳入对象年龄分布过于分散，结果可能存在偏倚，未来需进一步完善，继续探索肺腺癌抗肿瘤治疗过程中发生VTE的影响因素。另外，本研究的研究对象是在呼吸内科治疗的肺腺癌患者，以化疗作为主要治疗手段，代表性有限，靶向治疗、放疗与肺腺癌VTE发生风险的关系有待进一步深入研究。

**Competing interests**

The authors declare that they have no competing interests.
